# STAT1 gain-of-function in 12 Moroccan patients: Clinical and genetic insights

**DOI:** 10.70962/jhi.20250021

**Published:** 2026-08-19

**Authors:** Bouchra Baghad, Ibtihal Benhsaien, Fatima-zahra El Fatoiki, Hind Ouair, Abderrahmane Moundir, Abderrahmane Errami, Maha Soussi-Abdellaoui, Mélanie Migaud, Jean-Laurent Casanova, Anne Puel, Jalila El Bakkouri, Ahmed Aziz Bousfiha, Soumiya Chiheb, Fatima Ailal

**Affiliations:** 1 Laboratory of Clinical Immunology, Infection and Allergy, Faculty of Medicine and Pharmacy of Casablanca, Hassan II University, Casablanca, Morocco; 2 Department of Dermatology and Venerology, CHU Ibn Rochd, University Hassan II, Casablanca, Morocco; 3 Clinical Immunology Unit, Department of Pediatrics, CHU Ibn Rochd, University Hassan II, Casablanca, Morocco; 4 Department of Parasitology and Mycology, CHU Ibn Rochd, University Hassan II, Casablanca, Morocco; 5 Laboratory of Human Genetics of Infectious Diseases, Necker Branch, INSERM UMR1163, Paris, France; 6 Imagine Institute, Université Paris Cité, Paris, France; 7 St. Giles Laboratory of Human Genetics of Infectious Diseases, Rockefeller Branch, The Rockefeller University, New York, NY, USA; 8 Howard Hughes Medical Institute, New York, NY, USA; 9 Immuno-serology Laboratory, Ibn Rochd University Hospital Center, Casablanca, Morocco

## Abstract

Heterozygous gain-of-function (GOF) variants in STAT1 are the most frequent genetic cause of chronic mucocutaneous candidiasis (CMC). Data from Africa remain scarce. We conducted a cross-sectional study at Ibn Rochd University Hospital, Casablanca (2016–2021), enrolling patients with persistent or recurrent mucocutaneous and/or invasive fungal disease. Whole-exome and/or Sanger sequencing were performed, with pathogenicity assessed using ACMG/AMP criteria. We identified 12 patients from 11 kindreds carrying 10 distinct heterozygous STAT1 GOF variants. CMC was the dominant manifestation (11/12, 91%). Mycobacterial infections were documented in 42% of patients and bacterial infections in 50%; one patient presented with cryptococcal meningitis. Autoimmunity was observed in three patients. Two patients died. The c.194A>G (p.D65G) variant, found in a father–son pair, may represent the first clinical description of this substitution as a STAT1 GOF variant. This first dedicated Moroccan cohort demonstrates substantial allelic heterogeneity and a clinical phenotype dominated by infections, particularly tuberculosis, with less frequent autoimmune manifestations than in Western cohorts.

## Teaser

STAT1 gain-of-function mutations cause chronic mucocutaneous candidiasis and broad immune dysregulation, yet African data are limited. In a Moroccan cohort, Baghad et al. describe clinical and genetic features of 12 patients, highlighting infectious predominance, phenotypic diversity, and the need for improved molecular diagnosis and targeted therapies.

## Introduction

Chronic mucocutaneous candidiasis (CMC) is a rare condition marked by persistent or recurrent *Candida *infections of the skin, nails, and mucous membranes, most often due to *Candida albicans* (1). It may appear as an isolated phenotype or in association with syndromic inborn errors of immunity (IEIs) (2, 3). Alongside chronic candidiasis, patients may experience recurrent staphylococcal or respiratory bacterial infections, chronic herpesvirus disease, superficial dermatophyte infections, and occasionally invasive fungal infections (4, 5). Some syndromic forms are also associated with autoimmune manifestations, particularly autoimmune thyroid disease or hepatitis. Prolonged, uncontrolled CMC has been associated with an elevated risk of oral and esophageal squamous cell carcinoma and, in rare cases, intracranial aneurysms (5). CMC is now recognized to result from selective defects in IL-17–dependent mucocutaneous immunity (6). Monogenic defects such as IL-17RA, IL-17RC, ACT1 (TRAF3IP2), RORC, and CARD9 deficiencies impair Th17 and innate lymphoid cell responses, and neutralizing autoantibodies against IL-17A, IL-17F, and IL-22 in AIRE deficiency produce a similar phenotype, establishing CMC as a key clinical entry point for diagnosing specific IEIs (6, 7, 8).

Signal transducer and activator of transcription 1 (STAT1) is a central transcription factor activated downstream of type I, type II, and type III interferons, as well as several interleukins, where it coordinates antiviral, antimycobacterial, and inflammatory responses (9). Germline STAT1 gain-of-function (GOF) variants cause an autosomal-dominant immunodeficiency that represents the most frequent genetic etiology of CMC worldwide (9). Since the initial description of STAT1 GOF mutations in patients with CMC in 2011 (10), at least 660 affected individuals and more than 135 distinct germline variants have been reported (11, 12, 13). These variants impair nuclear dephosphorylation of STAT1 and prolong its activation after interferon stimulation, resulting in exaggerated transcription of interferon-stimulated genes (14). Patient T cells show enhanced responsiveness to interferon-α and interferon-γ but a marked reduction in Th17 differentiation and in the production of IL-17A, IL-17F, and IL-22, providing a mechanistic explanation for the paradox of interferon-driven inflammation with defective mucocutaneous antifungal immunity (14). Experimental work further indicates that STAT1 GOF also disrupts epithelial barrier responses and mucosal cytokine networks, further compromising local control of *Candida *and other pathogens at mucosal surfaces (15).

Despite this expanding global literature, data from African populations remain scarce, and the contribution of STAT1 GOF to CMC and invasive fungal disease on the continent is poorly defined (11, 13). Existing reports from the Middle East and North Africa (MENA) indicate that IEIs are underdiagnosed and often detected late, particularly when they present with nonspecific chronic infections such as CMC (7). To date, only isolated STAT1 GOF cases have been described from the MENA region (16, 17, 18, 19, 20), and no dedicated cohort has been described from Morocco, leaving regional patterns of disease expression, genotype distribution, and access to advanced therapies such as JAK inhibition or hematopoietic stem cell transplantation (HSCT) largely unexplored (21, 22, 23, 24). Against this background, we aimed to characterize the clinical, immunological, and genetic features of Moroccan patients with chronic fungal disease who were found to carry heterozygous STAT1 GOF variants, in order to place this national experience within the broader international landscape of STAT1-associated IEIs.

## Results

### Epidemiological characteristics

We identified 12 Moroccan patients with chronic fungal infections carrying heterozygous GOF variants in STAT1. These patients originated from 11 unrelated kindreds, including one multiplex family comprising a father and his son (P8 and P7, respectively). The cohort included seven males and five females (male-to-female ratio, 1.4). Parental consanguinity was reported in two of the 11 families. At the time of last evaluation, patients’ ages ranged from 4 to 55 years. Nine patients were pediatric cases (ages 4–15 years; mean age 10.1 years [*n* = 9]), while three were adults (ages 30–55 years; mean age 38.3 years [*n* = 3]). The mean age at onset of CMC manifestations was 4 years (range, 3 mo to 15 years). In most patients, fungal disease began in infancy or early childhood, whereas two patients (P7 and P8) developed mucocutaneous candidiasis during adolescence, with onset at 15 years of age (Table 1).

**Table 1.** Clinical characteristics of Moroccan patients with STAT1 GOF variants

Patient	Sex	Age (y)	Age onset (y)	Consang	Chronic mucocutaneous fungal infections	Other infections	Autoimmune/inflammatory	Outcome
P1	M	7	0.3	No	Oral candidiasis, cutaneous mycosis, onychomycosis	Recurrent pneumonia, recurrent bacterial otitis	None	Alive, stable
P2	F	10	5	No	Oral and cutaneous candidiasis, cheilitis	Recurrent tonsillitis	None	Alive, stable
P3	M	13	0.3	No	Oral candidiasis, onychomycosis	None	Anemia, celiac disease	Alive, growth delay
P4	F	12	3	No	Tinea capitis, cutaneous and oral candidiasis, onychomycosis	Purulent otorrhea	Eczema, Raynaud’s phenomenon	Lost to follow-up
P5 (18)	M	30	2	Yes	Cutaneous dermatophytosis, oral candidiasis, onychomycosis	HSV gingivostomatitis, staphylococcal abscesses, TB hepatic abscess	Eczema, arthralgia	Alive
P6	M	42	5	No	None	Recurrent pulmonary TB, oral aphthosis	None	Alive, chronic lung disease
P7	M	15	15	Yes	Mild oral candidiasis	Cryptococcal meningitis, TB lymphadenitis	None	Alive, neurological recovery
P8	M	55	1	Yes	Onychomycosis, intertrigo, oral candidiasis	Pleuropulmonary TB, chronic warts (HPV)	None	Alive, postsurgical
P9	F	6	0.25	No	Cutaneous and oral candidiasis	None	None	Deceased (SCLS)
P10	F	4	1	No	Oral candidiasis, onychomycosis, tinea	Recurrent pneumonia (*S. pneumoniae*)	None	Deceased (anaphylaxis)
P11 (19)	M	5	0.3	No	Oral candidiasis, onychomycosis, intertrigo, tinea	BCGitis, herpetic gingivostomatitis, demodicosis	Bilateral blepharitis	Alive, stable
P12 (20)	F	12	0.5	Yes	Oropharyngeal candidiasis, onychomycosis	Recurrent furunculosis, staphylococcal abscess, demodicosis	Bilateral blepharitis	Alive, stable

M, male; F, female; HSV, herpes simplex virus; TB, tuberculosis; BCG, bacillus Calmette–Guérin; SCLS, systemic capillary leak syndrome; HPV, human papillomavirus; *S. pneumoniae*, *Streptococcus pneumoniae*. Patients P5 (18), P11 (19), and P12 (20) were previously reported as individual case reports.

### Clinical features

**Figure 1.**
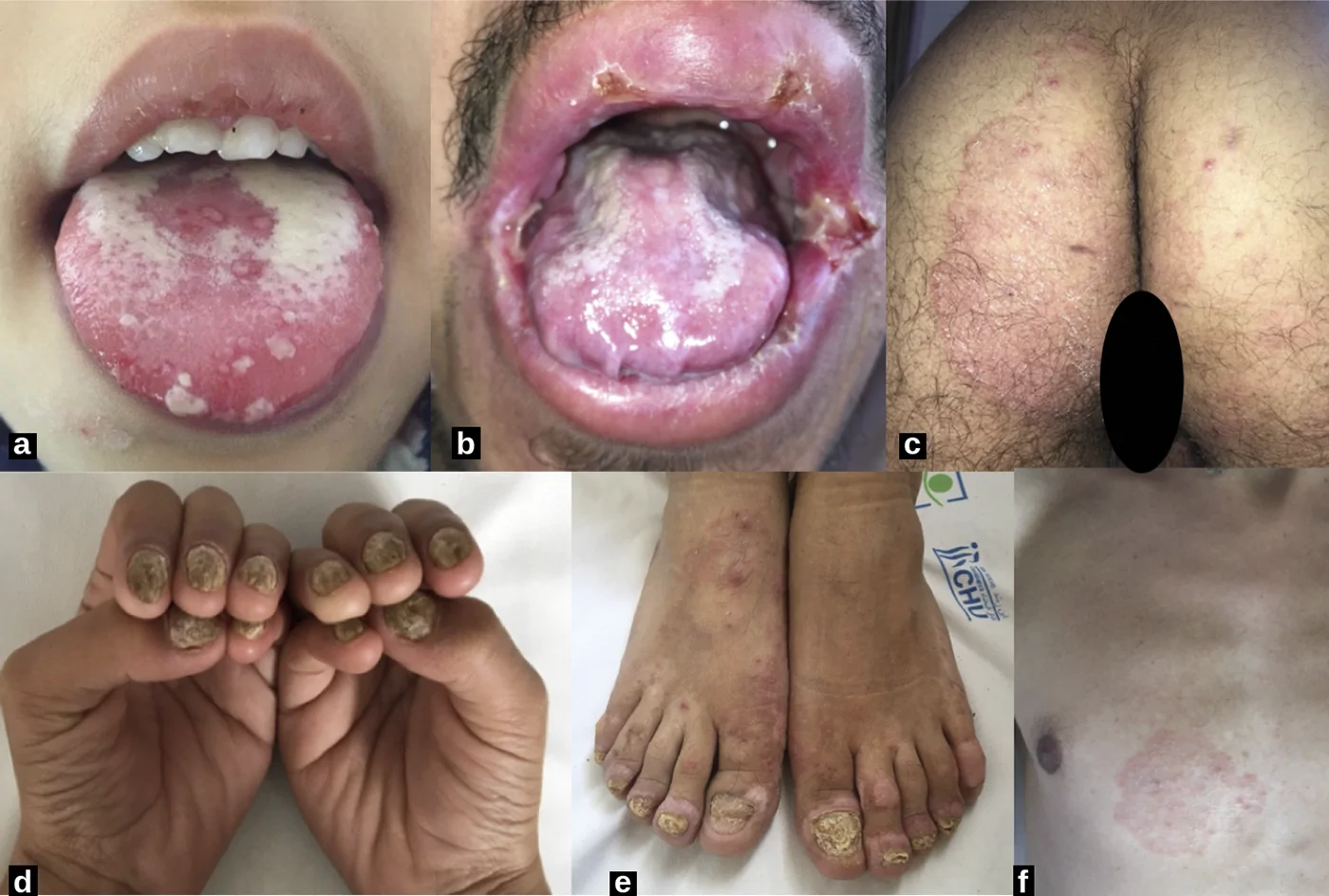
**Clinical photographs of patients with CMC. (a)** Oral thrush showing white pseudomembranous plaques on the tongue. **(b)** Gingivostomatitis with extensive mucosal involvement. **(c and f)** Diffuse cutaneous mycosis. **(d and e)** Onychomycosis with nail dystrophy affecting multiple digits. All photographs are anonymized and published with informed consent.

The clinical characteristics of the 12 patients carrying STAT1 GOF variants are summarized in Table 1. Chronic mucocutaneous fungal disease represented the dominant clinical presentation. Oral and/or oropharyngeal candidiasis was reported in 11 of the 12 patients, ranging from typical thrush (Fig. 1 a) to extensive mucosal involvement including episodes of gingivostomatitis (Fig. 1 b). Cutaneous fungal manifestations were also frequent, with diffuse cutaneous mycosis (Fig. 1, c and f) and intertriginous involvement documented in selected patients (P8, P11). Onychomycosis constituted a prominent and often long-standing feature, affecting 7 of 12 patients (Fig. 1, d and e). Dermatophyte infections were recorded in several cases, including tinea capitis (P4) and other tinea/dermatophytosis presentations (P5, P10, and P11). Patient P6 did not exhibit chronic candidiasis but experienced only transient episodes of stomatitis.

Infectious manifestations beyond fungal disease were heterogeneous (Table 1). Bacterial infections included recurrent pneumonia (P1, P10), recurrent tonsillitis (P2), and otologic infections, notably recurrent bacterial otitis (P1) and purulent otorrhea (P4). Staphylococcal skin infections were documented in two patients (P5 and P12). Mycobacterial infections were recorded in five patients and comprised recurrent pulmonary tuberculosis (P6), pleuropulmonary tuberculosis (P8), a tuberculous hepatic abscess (P5), tuberculous lymphadenitis (P7), and a localized bacillus Calmette–Guérin (BCG)–related infection (BCGitis) in one child (P11). One patient (P7) presented with cryptococcal meningitis as the initial manifestation of disease. Viral and parasitic infections included herpetic gingivostomatitis (P5, P11), chronic hand and foot warts related to human papillomavirus (HPV) infection (P8), and cutaneous demodicosis (P11, P12).

Autoimmune and inflammatory manifestations were variably observed and included anemia associated with celiac disease (P3), eczema associated with Raynaud’s phenomenon (P4), eczema with arthralgia (P5), and bilateral blepharitis (P11, P12). Several patients developed major complications reflecting both the chronic burden of mucocutaneous disease and systemic involvement. These included growth retardation in 4 of 12 patients (P3, P4, P9, and P10), psychomotor delay (P3), candidal esophageal stricture with progressive dysphagia (P4), thoracic kyphosis (P5), pulmonary sequelae following tuberculosis (P6), neurological involvement after cryptococcal meningitis (P7), and squamous cell carcinoma of the lower lip diagnosed at the age of 52 years (P8).

### Microbiological profile

The spectrum of microbiologically documented infections is summarized in Table 2. *C. albicans* was the most frequently isolated fungal pathogen, accounting for 14 infection episodes across the cohort. Other *Candida *species were less common, with *Candida dubliniensis* identified in two patients and *Candida krusei* in one. Dermatophyte infections, predominantly due to *Trichophyton *species, were confirmed in seven episodes of tinea capitis or tinea corporis. One episode of invasive fungal infection was documented, consisting of cryptococcal meningitis caused by *Cryptococcus neoformans* in P7. Among bacterial infections, *Staphylococcus aureus* was the most frequently isolated pathogen, responsible for five skin or soft-tissue infections. *Streptococcus pneumoniae* was identified in one episode of bacterial pneumonia, and *Enterococcus faecium* was isolated from a chronic wound infection. A total of five mycobacterial infections were recorded, including four patients with microbiologically confirmed *Mycobacterium tuberculosis* infections (P5–P8), and a child (P11) with localized BCG-related infection. Parasitic infection with Demodex mites (cutaneous demodicosis) was observed in two patients. With respect to viral infections, two episodes of severe herpes simplex virus type 1 infection were documented, and one patient presented with extensive cutaneous warts related to HPV infection. No episodes of clinically significant disease requiring hospitalization due to varicella-zoster virus, cytomegalovirus, or Epstein-Barr virus were observed in this cohort.

**Table 2.** Microbiological profile of patients with STAT1 GOF variants

Pathogen type	Specific pathogen	*n* (episodes)
Fungal	*C. albicans*	14
Fungal	*C. dubliniensis*	2
Fungal	*C. krusei*	1
Fungal	Dermatophytes (e.g., *Trichophyton*)	7
Fungal	*C. neoformans*	1
Bacterial	*S. aureus*	5
Bacterial	*S. pneumoniae*	1
Bacterial	*E. faecium*	1
Mycobacterial	*M. tuberculosis*	4
Mycobacterial	BCG strain	1
Parasitic	Demodex mite (folliculitis)	2
Viral	HSV-1	2
Viral	HPV	1

HSV-1, herpes simplex virus type 1. Episodes refer to microbiologically documented infections; multiple episodes may occur in the same patient.

### Immunological findings

Immunological testing was available for 11 of the 12 patients, and the corresponding data are presented in Table 3. Overall, serum immunoglobulin levels were within age-adjusted reference ranges (RRs) in most cases (25, 26). IgA was mildly decreased in P2 (0.45 g/L; RR 0.5–1.7 g/L), and IgG was below the RR in P2 (5.52 g/L; RR 6.2–11.5 g/L). Elevated IgG was noted in seven patients (P1, P3, P4, P8, P10, P11, and P12), with high values in P11 (18.1 g/L) and P8 (21.0 g/L) who received immunoglobulin replacement. IgM levels were within normal limits.

**Table 3.** Immunological findings in patients with STAT1 GOF variants

Patient	Age (y)	IgG (g/L)	IgA (g/L)	IgM (g/L)	IgE (IU/ml)	CD3^+^ (cells/μl)	CD4^+^ (cells/μl)	CD8^+^ (cells/μl)	CD19^+^ (cells/μl)	NK (cells/μl)
P1	7	11.74 ↑	1.13	0.90	5.25	1,428	779	560	442	91 ↓
P2	10	5.52 ↓	0.45 ↓	1.00	210 ↑	ND	ND	ND	ND	ND
P3	13	16.86 ↑	1.86	0.92	33.5	2,329 ↑	935	1,217 ↑	1,018 ↑	ND
P4	12	13.5 ↑	1.20	1.00	11.5	2,290	152 ↓	649	410	ND
P5	32	11	2	1.7	1.20	1,684	820	743	73	96
P6	42	13.24	3.30	0.03 ↓	0.67	1,463	544	754	444 ↑	146
P7	15	ND	ND	ND	ND	ND	ND	ND	ND	ND
P8	55	21.0 ↑	0.91	1.18	15.11	2,414 ↑	1,178 ↑	983 ↑	1,023 ↑	ND
P9	6	7.00	0.46	0.98	ND	2,000	800	768	753	10 ↓
P10	4	10.8 ↑	0.89	1.18	2.00	1,200 ↓	600 ↓	500	400	150
P11	10	18.1 ↑	7.20 ↑	3.90 ↑	649 ↑	2,002	923	768	753	10 ↓
P12	14	15.10 ↑	0.83	0.85	11.68	2,000	2,000 ↑	710	1,000 ↑	12 ↓

ND indicates not determined. ↑ indicates above and ↓ indicates below age-adjusted RRs. RRs were derived from the Primary Immunodeficiency Phenotypical Diagnosis App (25), based on International Federation of Clinical Chemistry and Laboratory Medicine pediatric reference data and lymphocyte subset reference values reported by Shearer et al. (2003) (26).

### Treatment and follow-up

Initially, all patients underwent a short-term antifungal treatment with oral fluconazole at 150 mg/day to manage the disease flare. Due to the absence of disease control, 10 patients were prescribed a long-term fluconazole cure (for at least 6 mo) with monthly monitoring of liver function tests. No clinical or biological adverse effects were noted in our patients with fluconazole. We identified one case of fluconazole resistance, which was subsequently replaced by voriconazole after fungal sensitivity testing. Patient P7, who presented with cryptococcal meningitis, received induction therapy with liposomal amphotericin B, followed by consolidation and long-term maintenance with fluconazole.

In addition to antifungal treatment, five patients (P1, P3, P6, P8, and P10) received cotrimoxazole prophylaxis for recurrent or severe bacterial infections. The two patients with demodicosis received topical metronidazole with a good outcome. Two patients (P1 and P11) received immunoglobulin replacement therapy for recurrent bacterial infections associated with functional antibody deficiency. Seven patients are stable to date. Two patients died: P10 following an acute anaphylactic reaction to a β-lactam antibiotic prescribed for a documented bacterial infection, and P9 from systemic capillary leak syndrome (SCLS), characterized by sudden hypotension, hemoconcentration, hypoalbuminemia, and generalized edema in the absence of identifiable infectious, septic, or vasculitic triggers.

### Genetic findings

All 12 patients carried heterozygous variants in STAT1, consistent with an autosomal-dominant GOF pattern (Table 4). In total, 10 distinct variants were identified in the cohort. Nine were missense single-nucleotide substitutions leading to amino acid changes in STAT1, and one was an intronic splice-region variant (c.541+63C>T in P2). Recurrent variants were observed: c.800C>T (p.A267V) was found in two unrelated patients (P3 and P6), and c.194A>G (p.D65G) in a father and his son (P8 and P7, respectively). The variants were distributed across several functional domains of STAT1, with the largest proportion located in the coiled-coil domain (4/10; 40%) and the N-terminal domain (NTD) (2/10; 20%), while fewer occurred in the DNA-binding domain, linker domain, and SH2 domain (each 1/10; 10%). Based on American College of Medical Genetics and Genomics (ACMG)/Association for Molecular Pathology (AMP) criteria (27), four unique variants were classified as pathogenic (c.1154C>T, c.800C>T, c.820C>T, and c.812A>C) and six as likely pathogenic (c.541+63C>T, c.1885C>T, c.194A>G, c.1633G>A, c.265A>T, and c.511G>A). All variants were absent or extremely rare in population databases, and in silico prediction (Combined Annotation Dependent Depletion [CADD]) supported a deleterious effect, with scores ranging from 15.0 to 33.0 (Table 4). No additional variants were identified in other known IEI-associated genes that could explain the clinical phenotype.

**Table 4.** Genetic findings in the 12 patients with STAT1 GOF variants

Patient	Variant (cDNA)	Protein	Type	Domain	ACMG	CADD	Previous reports
P1	c.1154C>T	p.T385M	Missense	DBD	Pathogenic	26.5	(5, 12, 13, 28)
P2	c.541+63C>T	–	Splice-region	–	Likely pathogenic	15.0	–
P3	c.800C>T	p.A267V	Missense	CCD	Pathogenic	24.7	(10, 13, 28, 29, 30)
P4	c.820C>T	p.R274W	Missense	CCD	Pathogenic	32.0	(13, 30)
P5	c.1885C>T	p.H629Y	Missense	SH2	Likely pathogenic	23.0	(18, 31, 32)
P6	c.800C>T	p.A267V	Missense	CCD	Pathogenic	24.7	(10, 13, 28, 29, 30)
P7	c.194A>G	p.D65G	Missense	NTD	Likely pathogenic	31.0	–
P8	c.194A>G	p.D65G	Missense	NTD	Likely pathogenic	31.0	–
P9	c.1633G>A	p.E545K	Missense	LD	Likely pathogenic	33.0	(33)
P10	c.265A>T	p.N89Y	Missense	NTD	Likely pathogenic	22.4	(11, 13)
P11	c.511G>A	p.D171N	Missense	CCD	Likely pathogenic	31.0	(11, 13, 19)
P12	c.812A>C	p.Q271P	Missense	CCD	Pathogenic	25.4	(13, 20, 30, 34)

NTD, N-terminal domain; CCD, coiled-coil domain; DBD, DNA-binding domain; LD, linker domain; SH2, Src homology 2 domain. ACMG classification according to Richards et al. (2015) (27).

## Discussion

To our knowledge, this Moroccan series represents one of the first dedicated descriptions of STAT1 GOF disease from Africa and provides cohort-level data from a region that remains severely underrepresented in the global literature on this IEI. Reported patient counts vary across publications depending on the time window and deduplication method applied: the 2021 systematic review by Zhang et al. identified 442 unique STAT1 GOF cases across 108 publications (11); a more recent mutation-level analysis integrating new reports with the historical international cohort assembled 533 unique patients from 36 countries carrying 135 distinct pathogenic mutations (12); and the 2024 comprehensive review by Guo et al. documented at least 660 affected individuals and more than 125 distinct germline variants (13). Among patients with documented country of residence (371 of 442 cases), the majority originate from Europe (48.2%), led by France (13.7%), Germany (9.7%), the United Kingdom (7.8%), the Netherlands (5.1%), and Italy (2.7%); North America (18.8%), principally the United States (12.9%), Mexico (3.5%), and Canada (2.4%); and Asia (13.5%), notably Japan (8.4%) and China (5.1%) (11). In contrast, epidemiological data from Africa (Morocco, 1.3%) and the Middle East, including Turkey (2.4%), Iran (1.1%), and Saudi Arabia (1.1%), remain markedly scarce (11). Ethnicity was specified for only 42 of those 442 patients, reflecting heterogeneous reporting practices in the primary literature rather than true epidemiological differences between populations (11). In this context, our series identifies 12 Moroccan patients from 11 kindreds carrying 10 distinct STAT1 GOF variants, supporting substantial allelic heterogeneity and suggesting that STAT1 GOF is likely underrecognized and underreported in our setting. Only one father–son pair (16.7%) represented a familial case, in contrast to the ∼61% of familial cases reported in large international series (5). Parental consanguinity was present in 18% of our kindreds, considerably higher than the 1.7–4.2% documented in larger global cohorts (11, 12). These findings highlight the importance of considering this diagnosis even in apparently sporadic cases.

CMC was the clinical hallmark in 91% (11/12) of our patients, a proportion consistent with the 92.8–98% prevalence documented in the largest published international cohorts (11, 12), and with the observation that CMC accompanies at least 85.6% of known STAT1 GOF mutation sites (13). Globally, the condition typically manifests in infancy or early childhood, with a reported median age of onset of ~1 year, and around 90% of affected individuals develop persistent fungal disease before the age of 10 years (11). In our cohort, the mean age at symptom onset was 4 years, ranging from 3 mo to 15 years of age, which is broadly consistent with this pattern. It is important to note that transient oral candidiasis is common among healthy infants and resolves spontaneously in the first months of life, so the key clinical feature warranting investigation is not the occurrence of candidiasis, but its persistence, severity, and recurrence despite conventional antifungal therapy (35). Despite this early onset, molecular diagnosis is frequently delayed; the mean diagnostic delay in STAT1 GOF averages 5.7 years (11), and the mean age of our patients at the time of study was 10.1 years for children and 38.3 years for adults, reflecting a prolonged delay that is a well-recognized challenge in regional IEI practice (35). One patient in our series (P7) presented initially with cryptococcal meningitis in adolescence, rather than CMC, a manifestation that is rare in STAT1 GOF globally, documented in only 12 cases within large published series (11). Other fungal agents may also be responsible for invasive infections to a lesser degree in this context, including *Pneumocystis jirovecii*, *Aspergillus *spp., and *Talaromyces marneffei* (11). Although CMC is nearly universal, one patient in our cohort (P6) and ∼2–7% of patients reported worldwide do not exhibit candidiasis at the time of evaluation (5, 11, 12). These cases serve as a critical reminder that STAT1 GOF can manifest without florid fungal infections and should be considered in any patient presenting with severe, unusual, or refractory mucocutaneous or invasive fungal disease.

The immunological basis of STAT1-GOF–associated CMC involves at least two complementary mechanisms. The canonical pathway centers on an imbalance between STAT1 and STAT3 signaling downstream of type I and type II interferons: hyperactivation of STAT1 suppresses the transcription of Th17-promoting genes and profoundly reduces the mucosal production of IL-17A, IL-17F, and IL-22, cytokines that are essential for antifungal barrier defense at mucosal surfaces (6, 14). A complementary model involves a mucosal type II interferonopathy (15, 36). In that study, genetic and pharmacologic inhibition of IFN-γ or JAK-STAT signaling ameliorated mucosal candidiasis, providing a rationale for JAK inhibitor therapy in CMC (37, 38). It should be noted, however, that the Break et al. findings were obtained in an AIRE deficiency model, and their direct applicability to STAT1 GOF disease has been debated; nevertheless, the consistent clinical response to JAK inhibitors in STAT1 GOF patients, which simultaneously restores IL-17 axis function and dampens mucosal interferon signaling, is compatible with this dual-mechanism model (37, 38, 39, 40). Taken together, the available evidence suggests that mucosal antifungal immunity in STAT1 GOF is undermined both by a systemic deficit in Th17 differentiation and by locally dysregulated interferon responses that impair epithelial defense independently of the Th17 count.

Beyond mucocutaneous fungal disease, our cohort displayed the broad infectious susceptibility that characterizes STAT1 GOF immune dysregulation. Clinically significant bacterial infections affected 50% of our patients, compared with ∼74% documented in international series (5). Lower respiratory tract infections were observed in 33.3% of our patients, compared with 47.5% reported worldwide (11, 12). Notably, our cohort exhibited a distinct regional shift toward mycobacterial disease, which was microbiologically confirmed in 41.6% of patients, a frequency that significantly exceeds the 6–10.3% prevalence documented in international series (5, 11, 12). This higher frequency likely reflects the high endemic burden of *M. tuberculosis* in Morocco and the national policy of routine neonatal BCG vaccination, consistent with the pattern of mycobacterial overrepresentation observed in other Moroccan IEI cohorts (41, 42). All 12 patients in our cohort received BCG at birth, and only one child (P11, 8.3%) developed localized BCGitis, a rate that falls within the 0–8% range reported in vaccinated STAT1 GOF populations (13), while four patients had confirmed *M. tuberculosis* infections. Two had pulmonary or pleuropulmonary disease (P6: recurrent pulmonary tuberculosis; P8: pleuropulmonary tuberculosis), and two had extrapulmonary manifestations (P5: tuberculous hepatic abscess; P7: tuberculous lymphadenitis). The susceptibility to mycobacteria in STAT1 GOF represents a genuine immunological paradox: despite hyperactive IFN-γ signaling, which is central to mycobacterial killing by macrophages, patients remain susceptible to mycobacteria, potentially due to the dysregulation of the IL-12/IL-23 pathways or the refractory effects of chronic IFN-γ exposure on macrophage-mediated immunity (23).

Viral manifestations, typically observed in roughly one third of global cases (5, 12), were infrequent and localized in our series, limited to recurrent herpes simplex stomatitis in two children and extensive HPV-related warts in one adult. These findings align with the established predisposition to cutaneous viral persistence in STAT1 GOF, driven by impaired Th1 and Th17 responses despite intact interferon signaling (13). Critically, we did not observe the severe systemic viral complications reported elsewhere, such as cytomegalovirus organ disease or progressive multifocal leukoencephalopathy; however, this should be interpreted cautiously as our cohort is younger than most published series and severe viral complications in STAT1 GOF increase with age. Demodicosis in the context of STAT1 GOF is rare, with fewer than 10 cases documented in the literature; its identification and successful management with topical metronidazole in our series suggest that unchecked Demodex proliferation may serve as a critical cutaneous diagnostic indicator for STAT1 GOF in pediatric patients (20).

Overt autoimmunity was infrequent in our series, in contrast to many published Western cohorts. Only one patient (P3) had a clearly documented autoimmune condition, namely, celiac disease confirmed by positive antitissue transglutaminase antibodies and associated anemia, and no patient had autoimmune thyroiditis, autoimmune cytopenias, or an IPEX-like syndrome. Eczema with Raynaud’s phenomenon and eczema with arthralgia were present in two further patients (P4 and P5), consistent with the atopic and inflammatory features reported in a subset of STAT1 GOF patients globally (5, 9, 11). Large international series report autoimmune manifestations in 51–62% of STAT1 GOF patients, most commonly thyroid disease, hepatitis, and cytopenias (11, 13). The low autoimmune burden in our series warrants cautious interpretation. Systematic screening for subclinical autoimmunity, including thyroid function tests, antithyroid antibodies, and other autoimmune markers, was not uniformly performed across all patients, and milder or asymptomatic forms of autoimmunity may therefore have been missed. Furthermore, the relatively young age of most patients in our cohort (mean 10.1 years for children) may not have provided sufficient follow-up time for autoimmune manifestations to emerge, given that thyroid disease in particular tends to increase in frequency with age at diagnosis (5, 11). Possible regional genetic modifiers could contribute as well, but were not formally assessed. Intracranial or aortic aneurysms were not detected in our cohort, but vascular imaging was performed only when clinically indicated. Published data indicate that the incidence of aneurysmal vascular disease in STAT1 GOF is approximately three times higher than in the general population, and aneurysm formation is more common in patients with underlying autoimmunity and carries a poor prognosis (5, 43). Routine vascular surveillance by magnetic resonance angiography is therefore advisable in all patients once the diagnosis is established.

Growth failure was observed in 4 of 12 children (P3, P4, P9, and P10), likely reflecting the cumulative metabolic and nutritional consequences of chronic infection and prolonged systemic inflammation, as reported in other STAT1 GOF series (11, 12, 13). One patient (P8) developed squamous cell carcinoma of the lower lip at the age of 52 years, after decades of inadequately controlled oral candidiasis. This finding is consistent with the overall cancer rate of ∼5.9% reported in STAT1 GOF (11), and with the hypothesis that chronic *Candida*-associated mucosal inflammation promotes epithelial dysplasia and malignant transformation through nitrosamine production and persistent inflammatory signaling (44). The role of STAT1 in tumor biology is complex, encompassing both tumor-suppressive and tumor-promoting effects depending on the cellular compartment and tumor microenvironment, but chronic mucosal infection and inflammation are the most plausible carcinogenic drivers in this specific clinical context (44, 45). Long-term oncological surveillance is therefore warranted in STAT1 GOF patients, particularly those with a history of severe or prolonged CMC.

The 10 distinct STAT1 variants identified in our cohort reflect the allelic heterogeneity that is typical of STAT1 GOF disease. 9 of the 10 variants have been previously reported in published STAT1 GOF patients or in curated variant databases; the intronic splice-region variant c.541+63C>T identified in patient P2 has not been clearly documented in prior reports and may represent a novel variant (11, 12, 13). The variants were distributed across multiple STAT1 functional domains, with the highest concentration in the coiled-coil domain (4/10, 40%) and the NTD (2/10, 20%), in keeping with the known predilection of recurrent mutations for these regions in international cohorts (11, 12, 13). Two patients (P3 and P6) shared the recurrent c.800C>T (p.A267V) variant, which is among the most frequently reported STAT1 GOF mutations worldwide and has been associated with pulmonary complications including bronchiectasis (10, 13, 28, 29, 30). The p.R274W variant (P4) has similarly been linked to an elevated risk of respiratory involvement in some series (11, 13, 30). The p.T385M variant (c.1154C>T, P1) has recently been reported to confer among the most severe disease outcomes, with elevated risks of lower respiratory tract infections, aneurysm formation, and malignancy (12). The p.D65G (c.194A>G) variant, shared by P8 (father) and P7 (son), has not been reported in any prior STAT1 GOF clinical series or variant database. The only published cases at this NTD residue describe p.D65A (c.194A>C) in a three-member family with CMC and, in two adults, eosinophilic esophagitis (46). Both p.D65A and an adjacent p.D66A variant have subsequently been confirmed as GOF variants (47). Neither P7 nor P8 had eosinophilic esophagitis; P7 presented with CMC, tuberculous lymphadenitis, and cryptococcal meningitis, and P8 with CMC and pleuropulmonary tuberculosis. The variant (p.D65G) was absent from population frequency databases, with a CADD score of 31.0, supporting its classification as likely pathogenic. Parental segregation analysis, performed when DNA samples were available, confirmed a de novo origin for the c.812A>C (p.Gln271Pro) variant in patient P12, who had both parents tested with negative results. For the remaining patients, parental DNA was not systematically available, precluding formal de novo assessment. No additional pathogenic or likely pathogenic variants in other IEI-associated genes were identified in any patient, supporting STAT1 GOF as the sole genetic diagnosis in all 12 cases.

The immunological profile of our cohort was broadly consistent with published STAT1 GOF immunophenotypes, though several individual abnormalities merit comment. Most patients had preserved serum immunoglobulin levels, although a mildly reduced IgA was noted in P2 and elevated IgE was present in P2 and P11, the latter in the context of prominent atopic features including eczema and bilateral blepharitis. Polyclonal IgG elevation was observed in several patients, a well-recognized feature of chronic immune stimulation in STAT1 GOF (5, 11). Increased absolute B cell counts were observed in P3 and P8, possibly reflecting secondary hyperactivation of the humoral compartment in the context of chronic immune stimulation. Two patients (P1 and P11) received immunoglobulin replacement therapy on the basis of recurrent infections associated with functional antibody deficiency, a qualitative humoral deficit that has been documented in STAT1 GOF patients who nonetheless maintain normal total IgG concentrations (48). Formal Th17 cell quantification and in vitro cytokine stimulation assays measuring IL-17A, IL-17F, and IL-22 production were not performed systematically in this cohort, which represents a recognized limitation of our study, since such assays would have provided direct functional evidence of the STAT1 GOF phenotype and reinforced the pathogenicity assignment of the identified variants.

Current management of STAT1 GOF disease is based on long-term antifungal prophylaxis, antibiotic prophylaxis for recurrent bacterial infections, immunoglobulin replacement for selected patients with functional humoral deficiency, and, increasingly, JAK inhibitors (now used for autoimmune complications and refractory CMC, not only severe disease) or HSCT (49). In the largest published series, azole resistance was identified in 39% of patients receiving continuous antifungal therapy and in 15% of those treated intermittently, with azoles being the most commonly implicated class (5). In our cohort, long-term fluconazole was prescribed in 10 of 12 patients and was generally well tolerated, with no clinical or biological adverse effects noted. Only one patient (P4) developed clinically significant fluconazole resistance, which was subsequently managed with voriconazole following fungal susceptibility testing. Patient P7, who presented with cryptococcal meningitis, received induction therapy with liposomal amphotericin B in accordance with standard guidelines for cryptococcal disease in the context of underlying immunodeficiency, followed by consolidation and long-term maintenance with fluconazole. Five patients received cotrimoxazole prophylaxis because of recurrent and severe bacterial infections, and two patients (P1 and P11) received immunoglobulin replacement for functional antibody deficiency. None of our patients received JAK inhibitor therapy during the study period. This was primarily due to limited availability and cost in our resource-constrained setting; access to advanced targeted therapies remains a critical unmet need in this region. The evidence base for JAK inhibitors, primarily ruxolitinib, is nevertheless growing: among 20 patients receiving JAK inhibitor therapy in the series reviewed by Zhang et al., 12 showed symptomatic improvement (11), and a larger multicenter European Society for Immunodeficiencies/European Society for Blood and Marrow Transplantation-Inborn Errors Working Party retrospective study of 45 STAT1 GOF patients on JAK inhibitor therapy reported partial or complete clinical improvement in 87% of cases (50). Subsequent case series and a pediatric cohort analysis have further confirmed partial-to-marked resolution of CMC, alopecia, and inflammatory manifestations, often accompanied by partial restoration of IL-17 responses (37, 38, 39). Responses are variable, however, and relapse may occur after treatment interruption; careful infectious monitoring is also required given the risk of bacterial, fungal, and viral complications associated with JAK inhibition (40).

Concerning HSCT, early published data in STAT1 GOF were unfavorable: of 25 transplanted patients in the Zhang et al. series, 10 died, though seven achieved resolution of disease-related symptoms and four showed immune reconstitution (11). More recent reports suggest that outcomes are improving with better patient selection, optimized conditioning regimens, and, in some cases, pretransplant bridging with JAK inhibitors, such that HSCT is now increasingly considered for patients with severe, progressive, or treatment-refractory disease (11, 21, 49). Two patients in our cohort died during the study period. Patient P10 died following an acute anaphylactic reaction to a β-lactam antibiotic prescribed for a documented bacterial infection, a treatment-related adverse event rather than a direct complication of STAT1 GOF itself. Patient P9 died from SCLS, which occurred in the absence of identifiable infectious, septic, or vasculitic triggers at the time of the episode. Whether SCLS in this patient was causally related to immune dysregulation from the underlying STAT1 GOF variant or represented an independent event remains uncertain. These outcomes highlight the vulnerability of STAT1 GOF patients over time and the need for sustained, multidisciplinary follow-up.

Several limitations of this study should be acknowledged. The cohort is relatively small (*n* = 12) and was assembled through a single-center, phenotype-driven diagnostic workflow, which introduces ascertainment bias and does not allow unbiased estimation of the prevalence of STAT1 GOF among all patients with chronic mucocutaneous fungal disease in Morocco. Systematic screening for subclinical autoimmunity, including routine thyroid function testing and autoimmune serology, was not uniformly applied, and it is possible that mild autoimmune manifestations went undetected. Routine cerebrovascular imaging was not performed, so the absence of detected aneurysms cannot be taken as evidence of protection from this complication. Species-level identification was not consistently available for dermatophyte isolates, and formal Th17 cell quantification and cytokine production assays were not carried out systematically.

### Conclusions

This study represents the first dedicated cohort-level description of STAT1 GOF disease from Morocco and, more broadly, from Africa. The allelic diversity observed, with 10 distinct variants among 12 patients, points to a broader prevalence in Moroccan and African populations than the sparse literature suggests. The rarity of reported cases most likely reflects underdiagnosis and limited access to genetic testing rather than a true low burden of disease in the region. The clinical phenotype was dominated by CMC and infectious complications, with a mycobacterial disease rate of 42%, well above published international figures, in keeping with the high endemic tuberculosis burden in Morocco and the national BCG vaccination policy. Autoimmune manifestations were less prominent than in Western cohorts, though systematic screening was incomplete and the cohort was young. These findings emphasize that STAT1 GOF should be considered in patients with persistent or refractory mucocutaneous fungal disease, particularly when associated with mycobacterial or unusual infections. Early molecular diagnosis enables timely surveillance for serious complications, including esophageal stricture, cerebral aneurysm, and malignancy. Rapid access to immunological and genetic testing, together with improved availability of JAK inhibitor therapy and HSCT in resource-limited settings, is essential to improve outcomes in this setting.

## Materials and methods

### Patients and study design

We conducted a cross-sectional, prospective study at the Ibn Rochd University Hospital Centre in Casablanca between 2016 and 2021. Patients were recruited through the Department of Dermatology and Venereology and the Clinical Immunology Unit of the Department of Pediatrics. We included all individuals, without age or sex restriction, who presented with persistent or recurrent clinical manifestations compatible with CMC or invasive fungal disease of the skin, nails, and/or mucous membranes, but also severe dermatophyte infections or other deep mycoses. All patients carrying a pathogenic or likely pathogenic STAT1 GOF variant were enrolled; patient P6, who lacked overt CMC but carried a confirmed STAT1 GOF variant, was included on the basis of genotype. Persistent CMC was defined as continuous symptoms lasting ≥6 mo despite appropriate therapy, and recurrent CMC as ≥3 clinically documented episodes per year. We excluded patients with acquired causes of immunodeficiency, in particular those with documented HIV infection or receiving systemic immunosuppressive therapies, as well as patients with an identified IEI, from further investigation. All enrolled patients underwent a comprehensive dermatological and systemic examination. The distribution and severity of mucocutaneous candidiasis (oral, cutaneous, or ungual) and other fungal infections, such as dermatophytosis or invasive fungal disease, were recorded. Epidemiological and clinical data were prospectively collected for all patients using a standardized form, including age, sex, geographic origin, age at onset of symptoms, disease duration at diagnosis, pattern and severity of fungal disease, history of bacterial, viral, or parasitic infections, autoimmune or inflammatory manifestations, malignancies, vascular complications (aneurysms), growth parameters, and family history of similar symptoms or consanguinity.

### Immunological evaluation

All patients underwent a baseline immunological workup at the time of enrollment, as part of the initial diagnostic evaluation. This included serum immunoglobulin levels (IgG, IgA, IgM, and IgE), lymphocyte subset enumeration by flow cytometry (T cells, with CD4^+^ and CD8^+^ subsets; B cells; and natural killer [NK] cells), and screening for other immune defects.

### Genetic analysis

Genomic DNA was extracted from whole blood using standard procedures. Whole-exome sequencing and/or targeted Sanger sequencing were performed. Candidate variants in STAT1 identified by exome sequencing were confirmed by Sanger sequencing in index patients, and segregation analysis was carried out in available family members when possible. Sequence data were aligned to the reference human genome, and variants were annotated using standard bioinformatics pipelines. The pathogenicity of STAT1 variants was evaluated according to the ACMG/AMP guidelines (27), integrating population frequency data, predicted functional impact, segregation, previously published evidence, and clinical correlation. Variants were classified as pathogenic, likely pathogenic, benign, likely benign, or variant of uncertain significance. In silico prediction tools, including PHRED-scaled CADD scores, were used to support the assessment of novel or rare missense variants.

## Ethics approval

This study was conducted according to the principles specified in the Declaration of Helsinki and the local ethical guidelines. The protocol was approved by the Ethics Committee for Biomedical Research of the Faculty of Medicine and Pharmacy, University Hassan II of Casablanca, Morocco (International Review Board 00002504).

## Consent to participate

Written informed consent for participation and genetic analysis was obtained from all adult patients and from parents or legal guardians of minors, with assent from older children when appropriate.

## Consent for publication

The authors affirm that human research participants provided informed consent for publication of all data in this study, including specific consent for publication of anonymized clinical and photographic data.

## Data Availability

All data supporting the findings of this study are available within the main text. All STAT1 variants identified in this study have been deposited in ClinVar (accession numbers SCV007664802–SCV007664811). Additional data are available from the corresponding author upon reasonable request.
